# Machine learning-based prediction of one-year mortality in ischemic stroke patients

**DOI:** 10.1093/oons/kvae011

**Published:** 2024-11-14

**Authors:** Ahmad Abujaber, Said Yaseen, Yahia Imam, Abdulqadir Nashwan, Naveed Akhtar

**Affiliations:** Nursing Department, Hamad Medical Corporation (HMC), 3050 Doha, Qatar; School of Medicine, Jordan University of Science and Technology, 22110 Irbid, Jordan; Neurology Section, Neuroscience Institute, Hamad Medical Corporation (HMC), 3050 Doha, Qatar; Nursing Department, Hamad Medical Corporation (HMC), 3050 Doha, Qatar; Department of Public Health, College of Health Sciences, QU Health, Qatar University, 2713 Doha, Qatar; Neuroradiology Department, Neuroscience Institute, Hamad Medical Corporation (HMC), 3050 Doha, Qatar

**Keywords:** ischemic stroke, mortality, machine learning, early prediction, personalized medicine

## Abstract

Background: Accurate prediction of mortality following an ischemic stroke is essential for tailoring personalized treatment strategies. This study evaluates the effectiveness of machine learning models in predicting one-year mortality after an ischemic stroke. Methods: Five machine learning models were trained using data from a national stroke registry, with logistic regression demonstrating the highest performance. The SHapley Additive exPlanations (SHAP) analysis explained the model’s outcomes and defined the influential predictive factors. Results: Analyzing 8183 ischemic stroke patients, logistic regression achieved 83% accuracy, 0.89 AUC, and an F1 score of 0.83. Significant predictors included stroke severity, pre-stroke functional status, age, hospital-acquired pneumonia, ischemic stroke subtype, tobacco use, and co-existing diabetes mellitus (DM). Discussion: The model highlights the importance of predicting mortality in enhancing personalized stroke care. Apart from pneumonia, all predictors can serve the early prediction of mortality risk which supports the initiation of early preventive measures and in setting realistic expectations of disease outcomes for all stakeholders. The identified tobacco paradox warrants further investigation. Conclusion: This study offers a promising tool for early prediction of stroke mortality and for advancing personalized stroke care. It emphasizes the need for prospective studies to validate these findings in diverse clinical settings.

## BACKGROUND

Stroke represents a significant challenge to global health, ranking as the second leading cause of death worldwide and a principal cause of long-term disability [1]. The World Health Organization (WHO) reports that each year, about 13.7 million people experience a stroke, which results in around 5.5 million deaths due to related complications [1]. Moreover, stroke is a critical cause of enduring disability, with over half of the survivors aged 65 and above suffering from reduced physical mobility [2, 3].

Research in the stroke field achieved significant progress in determining the factors influencing stroke outcomes. Scholars have utilized various predictive models to estimate the likelihood of stroke mortality, including the Acute Physiology and Chronic Health Evaluation II (APACHE II) and the Sequential Organ Failure Assessment (SOFA). These models have proven effective in predicting outcomes, as evidenced by their accuracy and the area under the curve (AUC) metrics [4–6]. Additionally, logistic regression-based models have been widely applied to predict the outcome of strokes. A systematic review 2018 evaluating the effectiveness of different stroke prediction models based on logistic regression reported an overall satisfactory predictive performance, with a median AUC of 0.8. However, the requirement for more accuracy and precision in predicting stroke outcomes fuels the hunt to acquire more accurate and reliable prognostic tools [7].

In the past decade, the use of machine learning algorithms to predict stroke outcomes has significantly increased [8]. These algorithms have shown equal or superior effectiveness in predicting stroke prognosis across short-, medium-, and long-term outcomes, with many instances achieving accuracy and area under the curve (AUC) metrics exceeding 0.9 [9–11]. The impressive performance and strong predictive power of machine learning technologies are revolutionizing personalized healthcare, enabling customized treatments to meet each patient’s specific needs. Consequently, machine learning is swiftly adopted in the healthcare industry, underscoring its potential to transform patient care [8, 12].

**Figure 1:**
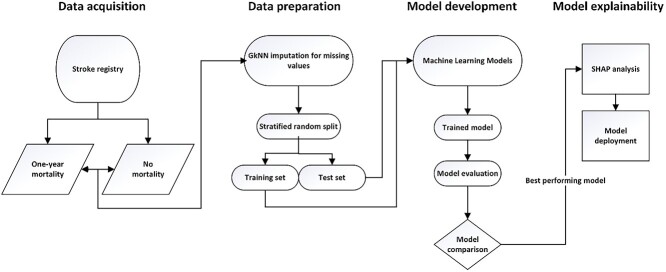
Flow diagram for the proposed ischemic mortality prediction system.

Recent studies have identified factors that were influential in forecasting stroke outcomes. These include demographic details of the patient, clinical characteristics, medical history, and treatment methodologies [13]. Crucially, aspects such as age, sex, Body Mass Index (BMI), and pre-stroke functional status—as evaluated through the pre-admission modified Rankin Score (mRS)—have emerged as significant determinants of stroke patients’ outcomes [14, 15]. Additionally, lifestyle choices, especially smoking habits, have been highlighted for their influence on recovery prospects, with evidence suggesting that non-smokers tend to have a better chance of favorable rehabilitation [16].

Clinical metrics are pivotal in predicting outcomes after a stroke. For instance, the size of the infarction directly correlates with clinical outcomes post-ischemic stroke, where smaller infarcts and better initial perfusion are linked to more favorable outcomes [17]. The National Institutes of Health Stroke Scale (NIHSS) scores post-thrombectomy, and the requirement for decompressive hemicraniectomy have been identified as significant predictors of mortality risk [14, 18]. The occurrence of hospital-acquired infections, including urinary tract infections and pneumonia, have been shown to influence prognosis, particularly mortality rates [4, 19]. Beyond clinical assessments and the severity of stroke upon admission, the presence of comorbid conditions—such as diabetes mellitus (DM), hypertension (HTN), dyslipidemia, previous stroke events, coronary artery disease (CAD), a history of atrial fibrillation (AF), or congestive heart failure (CHF)—were also found to predict disability and mortality [13, 14, 18, 20]. Furthermore, time-lapse from the onset of the stroke to hospital arrival timing, the doo-to-needle and door-to-groin time, and puncture to recanalization timings with lesser timing are associated with an enhanced probability of a positive prognosis [21, 22].

Qatar’s stroke scenario presents a unique case. Despite its economic wealth, the nation grapples with significant public health issues such as obesity, diabetes mellitus (DM), and cardiovascular diseases, with obesity affecting over 35% of the populace as of 2020 and diabetes mellitus affecting about 16% in 2013 [23, 24]. Surprisingly, Qatar has a relatively low stroke incidence rate of 58 cases per 100 000 people, significantly less than the MENA region’s average of 250 cases per 100 000 [25, 26], and maintains a low stroke mortality rate [25]. The demographic makeup, heavily skewed towards an expatriate workforce, plays a crucial role in shaping these statistics [27, 28]. The average stroke incidence among adults is 92.04 per 100 000, with the first stroke typically occurring around the age of 64. Ischemic stroke accounts for 73.7% of cases, primarily attributed to small vessel disease. Hypertension and diabetes are particularly widespread within this group, impacting 82.7% and 71.6% of stroke patients, respectively. Notably, Qatari females are at a higher risk of stroke-related disability or death within 90 days post-stroke compared to males, often experiencing strokes at older ages with higher rates of hypertension and diabetes [29].

Predicting mortality related to ischemic strokes is critically important, as it helps healthcare professionals personalize treatment strategies and allows patients and their families to set realistic expectations [30]. Therefore, this study aims to develop and evaluate a machine learning-based model to forecast the one-year mortality rates for ischemic stroke following the initial stroke event.

## MATERIAL AND METHODS

### Methods

Our proposed methodology involves a systematic approach to utilizing machine learning techniques to analyse data from a stroke registry. Initially, we gathered comprehensive data from the registry, ensuring the inclusion of all relevant variables. Following this, we conducted a thorough data analysis to uncover correlations among the variables. The dataset was then split for training multiple machine learning models. These models underwent evaluation, with the best-performing model selected for further analysis and potential deployment. Figure 1 outlines the sequential steps of our machine-learning prediction system.

### Ethical approval

The research obtained authorization from the institutional research board (IRB) at Hamad Medical Corporation, Qatar, under reference MRC-01-22-594.

### Data collection

Data were collected from the Stroke Registry at Hamad General Hospital (HGH) from January 2014 to July 2022. The dataset includes all patients aged 18 and above who were admitted to HGH with a primary diagnosis of stroke, covering ischemic stroke, transient ischemic attack (TIA), intracerebral hemorrhage (ICH), and stroke mimic. Since the establishment of the stroke registry in Qatar, a total of 15 859 patients have received specialized stroke care at the hospital.

### Baseline variables

Collected data covered a broad range of patient characteristics, including demographics, ethnic backgrounds, initial hemodynamic indicators (e.g. heart rate (HR) and blood pressure (BP)), risk factors for stroke, pre-existing medical conditions, site of admission, in-hospital outcomes such incidents of hospital-acquired infections (for instance, pneumonia and urinary tract infections), mortality rates, and the severity of the stroke at the time of admission. The stroke severity upon hospital entry was assessed using the National Institute of Health Stroke Score (NIHSS) [31, 32]. The modified Rankin Scale (mRS), which rates the patient’s pre-stroke functional status on a scale from 0 to 6, was also documented upon admission [9]. The ischemic stroke subtype was classified according to the Trial of Org 10 172 in Acute Stroke Treatment (TOAST) criteria [33].

Regarding ethnic background, the patients were divided into five groups based on their self-reported nationality: Qatari, from the Middle East and North Africa (MENA) region, from the South Asia region, from the South East Asia region (as defined by the United Nations geographical classification), and a collective category for all other nationalities labeled as ‘other’ [34–36]. The categorization of Qatari patients as a separate group was specifically designed to enable detailed comparisons, reflecting the unique demographic makeup of Qatar, where a significant portion of the populace consists of expatriates [28, 29]. This approach to categorization follows the precedent set by prior studies examining strokes in Qatar [34, 37]. Key risk factors, such as pre-existing medical conditions and smoking habits, were identified during hospitalization and confirmed by stroke registry staff through the examination of electronic medical records. These details are summarized in Table 1.

**Table 1:** Statistical characteristics of the collected stroke dataset.

Variable	Feature	Alive	Deceased	Total
Age	mean < 54.5	4121	85	4206
	mean ≥ 54.5	3760	217	3977
Sex	Male	6352	195	6547
	Female	1529	107	1636
Ethnicity	Qatari	1344	93	1437
	MENA	1458	74	1532
	South Asian	4079	105	4184
	South-East Asian	638	16	654
	Other	362	14	376
Mode of Arrival	Ambulance	5643	227	5870
	Other	2238	75	2313
Stroke Severity at Admission (NIHSS)	mean < 5.52	5619	71	5690
	mean ≥ 5.52	2262	231	2493
Onset of Symptoms	≤ 3 hours	2090	106	2196
	3–6 hours	1031	38	1069
	> 6 hours	4760	158	4918
GCS at Admission	mean < 14.4	784	143	927
	mean ≥ 14.4	5388	100	5488
Random Blood Sugar at admission (mmol/l)	mean < 9.4	5004	150	5154
	mean ≥ 9.4	2712	145	2857
Heart Rate at admission (bpm)	mean < 83	4144	135	4279
	mean ≥ 83	3625	160	3785
Systolic BP at admission (mmHg)	mean < 156	4267	208	4475
	mean ≥ 156	3590	90	3680
Diastolic BP at admission (mmHg)	mean < 91.4	4306	209	4515
	mean ≥ 91.4	3543	88	3631
Pre-stroke mRS	mean < 0.42	6871	168	7039
	mean ≥ 0.42	1010	134	1144
BMI	mean < 27.7	4048	161	4209
	mean ≥ 27.7	3117	112	3229
Diabetes Mellitus	No	3493	111	3604
	Yes	4388	191	4579
Hypertension	No	2264	88	2352
	Yes	5617	214	5813
Dyslipidemia	No	4252	197	4449
	Yes	3629	105	3734
Prior Stroke	No	6957	253	7210
	Yes	924	49	973
Atrial Fibrillation	No	7495	239	7734
	Yes	386	63	449
CAD	No	6946	230	7176
	Yes	935	72	1007
Tobacco Use	No	5967	273	6240
	Yes	1914	29	1943
Thrombolysis	No	7013	265	7278
	Yes	868	37	905
Thrombectomy	No	7577	284	7861
	Yes	304	18	322
Admission Location	Stroke Unit	3745	75	3820
	ICU	339	94	433
	Other	3797	133	3930
Hospital-acquired Pneumonia	No	7625	234	7859
	Yes	256	68	324
Hospital-acquired UTI	No	7705	276	7981
	Yes	176	26	202
Ischemic Stroke Subtype (TOAST)	LAA	1288	70	1358
	CE	895	67	962
	SAO	2899	36	2935
	SOC	767	17	784
	SUC	2032	112	2144
Platelet Count at admission	mean < 270	4189	173	4362
	mean ≥ 270	3415	112	3527
PT	mean < 11.3	4118	113	4231
	mean ≥ 11.3	2517	163	2680
APTT	mean < 28.1	4249	169	4418
	mean ≥ 28.1	2320	104	2424
One-Year Mortality	Yes	8183	302	8485

GCS (Glasgow Coma Scale) at admission, which assesses consciousness levels. RBS (Random Blood Sugar), HR (Heart Rate), SBP (Systolic Blood Pressure), and DBP (Diastolic Blood Pressure) are captured upon admission. mRS (modified Rankin Score) measures pre-stroke functional status. Patients’ conditions are further described by the presence or absence of DM (Diabetes Mellitus), HTN (Hypertension), CAD (Coronary Artery Disease), AF (Atrial Fibrillation), and Dyslipidemia. Stroke management interventions include Thrombolysis and Thrombectomy, and patients may be admitted to the ICU (Intensive Care Unit) or Stroke Unit. HAP (Hospital-acquired Pneumonia) and UTI (Urinary Tract Infection) are also monitored during hospital stays. The ischemic stroke subtypes follow the TOAST classification, including LAA (Large Artery Atherosclerosis), CE (Cardio-Embolism), SAO (Small Artery Occlusion), SOC (Stroke of Other Determined Cause), and SUC (Stroke of Undetermined Cause). Platelet count, PT (Prothrombin Time), and APTT (Activated Partial Thromboplastin Time)

### Outcome variable

The outcome focuses on the mortality rate within one year of being admitted to the hospital for a stroke. The admission date serves as the reference point (Day 0) for ischemic stroke, as recorded in the registry, while any mortality captured within 365 days from Day 0 is considered a one-year mortality.

### Inclusion/exclusion criteria

The study targeted all adults (aged 18 and above) diagnosed with ischemic stroke. Out of the original cohort of 15 859 patients, 8183 adults with an IS diagnosis were selected for analysis. The study excluded 7676 records of other conditions, including hemorrhagic stroke (1657), Transient Ischemic Attack (TIA) (1410), and stroke mimics (4609).

### Handling missing data and class imbalance

The Gray K-Nearest Neighbor (GkNN) strategy was utilized for imputing data to manage missing data values. This method considers imputed instances as observed data, which are then merged with complete instances for future rounds of imputation. The GkNN algorithm is adept at imputing data in heterogeneous datasets, covering numerical and categorical attributes. Further, by treating imputed values as observed data and iteratively integrating them into subsequent rounds, GkNN enhances data stability and accuracy. Drawing from gray system theory, it can effectively identify patterns in incomplete data, which is especially useful for datasets with substantial missing values. GkNN minimizes bias by using local neighbor comparisons to reflect natural data variability and adapts well to complex, non-normal distributions [38, 39]. The data revealed that the Glasgow Coma Score (GCS) had the highest rate of missing data at 21.5%, with Partial Prothrombin Time (APTT) and Prothrombin Time (PT) following at 16% and 15.5%, respectively. Table 1 displays the missing value percentages for each variable. The dataset showed a low mortality rate of 3.7%, highlighting a significant class imbalance between dead and alive cohorts. To mitigate this, random under-sampling (RUS) was applied to the majority class, creating a balanced dataset. This method randomly eliminates instances from the majority class until its size matches that of the minority class, effectively addressing class imbalance concerns for these analyses [40–44]. Additionally, to enhance the performance and efficiency of the models, feature scaling through data normalization was performed before training the machine learning models.

### Model training and evaluation

The dataset was divided into a training set (80%) and a test set (20%) via stratified random sampling. The training set was used to develop our models, whose performance was assessed on the test set. We trained five different machine learning models, including XGBoost, Random Forest (RF), Support Vector Machine (SVM), Classification and Regression Tree (CART), and Logistic Regression (LR).

A comprehensive set of performance metrics was employed to evaluate the effectiveness of the models, including accuracy, precision, recall, F1-score, log loss, the area under the receiver operating characteristic curve (AUC), Matthew’s correlation coefficient (MCC), Brier score, sensitivity, specificity, and Negative Predictive Value (NPV) [45–49]. These indicators are crucial for understanding how well each model can distinguish between positive cases (patients who died) and negative cases (patients who survived), considering the presence of class imbalance. The model that demonstrates the best overall performance, particularly with the highest F1-score, will be selected as the preferred model for further detailed analysis and potential future application.

### Model explainability

SHAP (SHapley Additive exPlanations) is a prominent tool for interpreting the outcomes of machine learning models. It provides detailed insights by assigning individual-level importance scores, known as SHAP values, to each feature, allowing for a deeper understanding of each feature’s impact on specific prediction results. SHAP values reveal the direction and magnitude of each feature’s influence, making it possible for domain experts to interpret how particular factors contribute to individual outcomes. Beyond individual predictions, SHAP offers global insights by aggregating SHAP values across multiple instances, which helps analysts observe feature importance trends and identify potential biases or patterns within the model. The tool ensures fair attribution even when features are correlated, maintaining a consistent and unbiased view of each feature’s contributions. This dual functionality, providing detailed and aggregated explanations, makes SHAP invaluable for increasing interpretability and accountability, particularly in decision-sensitive applications where understanding the ‘why’ behind predictions is essential [50, 51].

## RESULTS

Table 1 presents the characteristics of the study’s participants, who had an average age of 54.5 ± 13.5 years. About 80% of these individuals are male, with an average National Institutes of Health Stroke Scale (NIHSS) score of 5.52 ± 4. The most common IS subtype is Small Artery Occlusion (SAO), at 35.8%, followed by Stroke of Undetermined Cause (SUC), at 26%, and large artery atherosclerosis (LAA), at 17%.

### Evaluation of trained models’ performance

**Figure 2:**
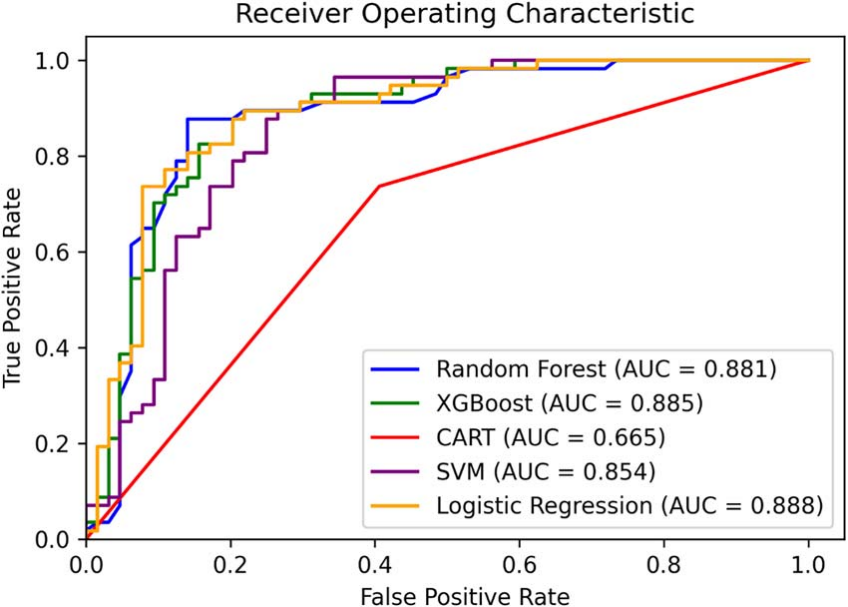
Area under the curve for the trained models.

The Logistic Regression model demonstrated remarkable performance with an accuracy of 83%, precision at 79%, a notable area under the curve (AUC) of 89%, and an F1-score of 83%, indicating high predictive accuracy. The Random Forest model showed comparable results, with an accuracy of 82%, precision of 76%, an AUC of 88%, and an F1-score of 82%. The performance of other models varied but did not reach the level of Logistic Regression. Based on the comprehensive performance indicators presented in Table 2 and Fig. 2, the Logistic Regression model emerged as the most effective and is selected for further SHAP analysis and potential future deployment.

**Table 2:** Machine learning models performance.

Model	Accuracy	Precision	Recall	F1 Score	Log Loss	Brier Score	AUC	Sensitivity	Specificity	NPV
Logistic Regression	0.83	0.79	0.88	0.83	0.46	0.14	0.89	0.88	0.79	0.88
Random Forest	0.82	0.76	0.89	0.82	0.46	0.14	0.88	0.89	0.76	0.90
XGBoost	0.80	0.74	0.89	0.81	0.60	0.15	0.89	0.89	0.74	0.90
CART	0.66	0.62	0.74	0.67	12.21	0.34	0.67	0.72	0.62	0.74
SVM	0.78	0.74	0.81	0.77	0.47	0.15	0.85	0.81	0.74	0.81

### SHAP analysis

**Figure 3:**
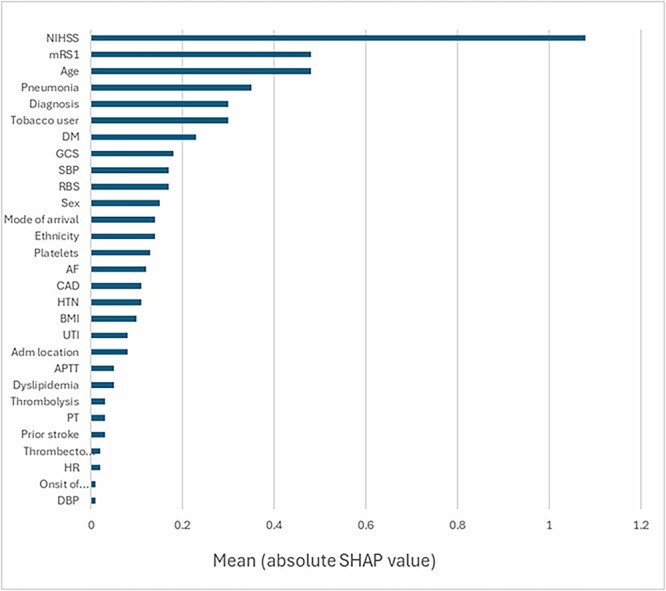
SHAP value for predictor importance.

The logistic regression model was selected for SHAP analysis to uncover the impact of various inputs on the model’s predictive capacity. The results indicated that the stroke severity at admission (NIHSS) had the greatest influence on the model’s predictions, followed by the pre-stroke functional status (mRS) as reported by family or caregivers, age, hospital-acquired pneumonia, a subtype of ischemic stroke (Dx), tobacco usage, and the history of diabetes mellitus (DM) (Fig. 3).

## DISCUSSION

This research evaluated the performance of five machine learning models and utilized SHAP analysis to identify the primary predictors of one-year mortality following an ischemic stroke. The logistic regression model emerged as the most effective compared to the others. The SHAP analysis of this model highlighted critical factors contributing to its predictive power, including the severity of the stroke as measured by the NIHSS score at admission, the pre-stroke functional status (mRS), age, the occurrence of hospital-acquired pneumonia, the subtype of ischemic stroke, tobacco consumption, and DM. Other variables had minimal impact on the model’s predictions, as indicated by their low SHAP values. Except for hospital-acquired pneumonia, a post-admission condition, all other significant predictors are assessable upon admission and can thus serve as early indicators of mortality risk. This study can improve clinical decision-making by enabling more accurate mortality predictions, allowing for more personalized care plans to mitigate preventable mortality risks. Additionally, it can help families set realistic expectations about the patient’s prognosis, thereby improving their engagement in the treatment process [52, 53].

Consistent with earlier studies, the severity, as measured by NIHSS, was identified as the predominant predictor of stroke prognostic outcomes, particularly mortality [18, 54]. Importantly, a significant difference was observed in the mean NIHSS scores between patients who passed away and those who survived (14.9 vs. 4.9), *P*-value < 0.05.

The pre-stroke functional status (mRS) was also identified as a significant factor in forecasting one-year mortality from ischemic stroke, aligning with existing literature that has incorporated the pre-stroke functional status (mRS) along with the modified stroke subtype, the Oxfordshire Community Stroke Project Classification, and age to create a mortality prediction tool (mSOAR). This tool is designed to predict 7-day in-hospital mortality and post-stroke disability and performs well [55, 56]. Importantly, it was found that the mean pre-stroke functional status (mRS) for deceased patients was significantly higher than for alive patients (1.5 vs. 0.37), *P*-valu < 0.05. Significantly, reports indicate that individuals with disabilities experience a higher mortality risk compared to those without disabilities. While this discrepancy can often be attributed to diseases and additional risk factors in cases of mild disability, it is essential to stress that we cannot rule out that more severe disabilities have an independent impact on mortality [57].

As seen in prior literature, there is a well-established correlation between higher age and increased stroke mortality [15, 18, 58]. This study’s mean age was 54.5 ± 13.5 years, which could be considered comparatively lower when assessed on regional and global scales [25]. The supplementary analysis showcased that the mean age of deceased patients was significantly higher than that of alive patients (64.4 vs. 54.4). Also, we found that 5.5% of patients surpassing the mean age had decreased, in contrast to 2% of others (*P*-value < 0.05), *P*-value < 0.05. While age itself is traditionally described as a non-modifiable risk factor for stroke and is associated with increased mortality, it is important to highlight the interaction between age and other potentially modifiable risks. Recent scholarly work has begun to view age as a potentially modifiable risk factor, distinguishing between biological and chronological age. This distinction has gained prominence in scientific research, highlighting an association between chronological age and the incidence of chronic diseases. However, recently, studies have clarified that this relationship is not directly causal [59].

The study also identified hospital-acquired pneumonia as a strong predictor of prognosis for ischemic stroke patients, who are susceptible to infections during their hospitalization, adversely affecting their recovery and prognostic outcomes, including mortality [60]. It was observed that 21% of patients developing hospital-acquired pneumonia passed away within a year of their stroke diagnosis, compared to only 3% of those without such pneumonia (*P*-value < 0.05). This highlights the critical need for healthcare providers to rigorously apply evidence-based preventative measures, including consistent oral hygiene, adjustments in posture to avoid aspiration, screening for dysphagia, and other interventions, as recommended by Grossmann and colleagues [61], to mitigate these risks.

Research indicates that the subtype of ischemic stroke significantly influences the stroke prognosis, including mortality [62, 63]. Our analysis revealed that patients with Small Vessel Occlusion (SVO) had the highest mortality rate at 7%, which is significantly higher than the 5.2% mortality rate observed in patients with large artery atherosclerosis (LAA) and stroke of undetermined cause (SUC), with a *P*-value < 0.05. Importantly, our findings suggest that the mortality risk for patients with SVO strokes is quadruple that of patients with other IS subtypes, emphasizing the severity of this stroke subtype in terms of mortality risk. This also coincides with previous reports that SVO is associated with worse prognostic outcomes, especially in high-risk populations [64]. In contrast, strokes resulting from cardioembolism (CE) were associated with the lowest mortality rate among IS subtypes, at just 1.2%.

The history of smoking surprisingly positively influences the one-year mortality rate following an ischemic stroke. Thus, individuals with a history of smoking had a lower mortality rate than non-smokers. Specifically, 1.5% of smokers passed away compared to 4.4% of non-smokers within a year of their ischemic stroke diagnosis, indicating statistical significance (*P*-value < 0.05). This counterintuitive result is referred to as the ‘tobacco paradox’ in stroke literature, where smokers seemingly have better outcomes than non-smokers [65]. Some explanations for this paradox suggest an age-related factor, encapsulated in the phrase, ‘the more you smoke, the earlier you stroke and the longer you have to cope’ [65]. Aligning with prior findings, our study noted that smokers had an average age of 51.4. At the same time, non-smokers were older on average at 55.8 years (*P*-value < 0.05), highlighting the intricate interplay between smoking history, age, and stroke outcomes.

The study highlights that having chronic comorbid conditions, such as diabetes mellitus (DM), not only elevates the risk of stroke occurrence but also adversely affects the prognosis of stroke, particularly in elderly patients [66, 67]. A significant correlation was found between diabetes and increased mortality rates, with diabetic patients experiencing a higher mortality rate compared to non-diabetic patients (4.2% vs. 3.1%), indicating statistical significance (P-value < 0.05). It is critical to note that the likelihood of death in diabetic patients is 1.43 times greater than in non-diabetic patients, underscoring the heightened risk of mortality associated with diabetes. This increase in mortality risk can be linked to the numerous irreversible damages diabetes inflicts on body tissues, which impede recovery and exacerbate disease outcomes. For instance, uncontrolled blood sugar levels lead to damage in blood vessels, resulting in extensive brain damage during a stroke. Additionally, diabetes contributes to microvascular damage within the brain, diminishing its ability to withstand and recover from stroke incidents, thereby heightening the risk of mortality [68–70].

Understanding the predictors of ischemic stroke mortality is immensely valuable in enhancing healthcare providers’ ability to deliver personalized medicine and improve patient outcomes. By identifying key factors such as stroke severity, pre-stroke functional status, age, comorbid conditions like diabetes, and even lifestyle factors like smoking history, clinicians can tailor interventions and management strategies more effectively. This knowledge empowers medical professionals to prioritize preventative measures, optimize treatment plans, and provide targeted rehabilitation services. Ultimately, leveraging these insights can lead to more accurate prognostications, better patient care, and improved survival rates, marking a significant step forward in pursuing personalized medicine in stroke care.

## LIMITATIONS

While the study utilizes national registry data, including all patients who received specialized stroke management in the country, several limitations should be considered when interpreting the study’s findings. First, the dataset was sourced exclusively from the stroke registry of a single institution, potentially limiting the generalizability of the findings to wider and more varied healthcare settings and patient demographics. The lack of specific data types, such as imaging findings and comprehensive laboratory test results, might have omitted important factors influencing stroke mortality prediction.

The study’s retrospective design inherently carries biases and limitations in capturing the nuances of clinical practice as it unfolds. Historical data may not reflect the latest advancements in treatment modalities or shifts in patient profiles, which could impact the model’s relevance to contemporary clinical scenarios. Furthermore, focusing on a one-year mortality prediction may overlook longer-term survival and health outcomes, reducing its applicability for longer-range prognostic evaluations.

Class imbalance, with a comparatively low number of mortality instances, posed additional challenges in model training and evaluation. Although under-sampling was employed to address this, the potential for model performance bias cannot be entirely dismissed.

The model demonstrated high accuracy and recall, yet the complexity inherent to machine learning approaches may hinder their straightforward interpretation and practical application in clinical environments.

Moreover, while SHAP analysis shed light on significant predictive factors, it did not establish causative links between these variables and stroke mortality. As such, while the study enhances our understanding of factors predictive of stroke mortality, its findings should be interpreted considering these limitations.

## CONCLUSION

This study developed a predictive model for ischemic stroke mortality using machine learning, achieving high accuracy by incorporating key predictors like NIHSS score severity, pre-stroke functional status, patient age, hospital-acquired pneumonia, ischemic stroke subtype, tobacco use, and diabetes presence. These factors allow for early risk identification and personalized clinical interventions. Despite its effectiveness, the model’s accuracy is limited by data quality issues and the exclusion of significant variables, emphasizing the need for broader variable inclusion. An unexpected finding was the tobacco paradox, suggesting lower mortality risk for smokers, highlighting the need for further research to validate the model’s clinical applicability. The study points towards personalized medicine, emphasizing predictive tools for patient-centered care and improved stroke management.

## Supplementary Material

OXFNSC-2024-003_R1_Review_history_kvae011

## Data Availability

The datasets generated and/or analyzed during the current study are available from the corresponding author on reasonable request and subject to appropriate ethical approvals.
