# Supplementary material for: Machine learning-based prediction of one-year mortality in ischemic stroke patients
Source: Oxf Open Neurosci. 2024 Nov 14;3:kvae011. doi: 10.1093/oons/kvae011 (PMC11576476; doi:10.1093/oons/kvae011)
Supplement: OXFNSC-2024-003_R1_Review_history_kvae011 [file oxfnsc-2024-003_r1_review_history_kvae011.zip › OXFNSC-2024-003_R1_Review_history_kvae011.odt]

Reviewer 1
Recommendation - Reject
The manuscript entitled "Machine Learning-Based Prediction of One-Year Mortality in Ischemic Stroke Patients" uses 5 machine learning models to predict the one-year mortality after ischemic stroke. 8183 adult stroke cases from Hamad General hospital, Qatar were included. 80% cases were used to train the machine learning, and 20% used for testing. Among the 5 models used, logistic regression achieved the best outcome with 83% success rate. Of the factors assessed, NIHSS, mRS, age, hospitalacquired pneumonia, small vessel stroke, diabetes, smoking were found to be associated with higher mortality. Although the approach is novel, the results are mostly expected. The only exception is smoking which is a known stroke risk factor. The patients included is possibly a reason for the outcome. There are 80% male stroke patients which is different from the global data with fewer male cases than female cases.
Qatar has a large population of migrant workers. Most of them are young male. This group of patients may lead to a younger age of stroke, usually with a better outcome. It is possible this group of patients has a higher rate of smoking.
Also there is a lack of discussion on thrombolysis and thrombectomy treatment on prognosis. These are important treatment for ischemic stroke. Even without a significance, there is a need to discuss the potential mechanisms.
Overall, this is an interesting study. But the clinical value is limited.

Review 2
Recommendation – Major Revision
In this paper, the authors explored the use of several machine learning algorithms to predict one-year mortality in ischemic stroke patients, employing SHAP to provide post-hoc model explanations. The clinical question is important, and the technical solution is straightforward. While the study has merits, necessary clarifications and revisions are needed.

In the Background section, the authors mentioned that scholars have developed and used models such as APACHE and SOFA to estimate the likelihood of stroke mortality. This statement requires adjustments, as neither score was specifically designed for stroke risk prediction.

Figure 1 requires further clarification. The links between several modules need to be clearly indicated and connected. For example, both training and test data are fed into the machine learning models; however, test data should be independently evaluated and therefore should be connected to model evaluation. Additionally, SHAP analysis is incorrectly linked to model comparison. SHAP is a post-hoc explanation tool that is separate from model comparison. The authors are encouraged to carefully examine the entire analytics pipeline to accurately reflect the work done.

A major concern in the methods section is the lack of clarity regarding the timestamps on variables available for prediction. To predict one-year mortality post-stroke, what is the time point of prediction? What is the timeframe for variable collection, e.g., pre-stroke or post-stroke within a specified number of months? Without a clear definition, there is a risk that some patients might use data from the 11th month post-stroke to predict one-year mortality (i.e., at the 12th month), while others may have only one month of post-stroke data available. The study design should be standardized and elaborated upon.

Missing value imputation, as shown in Figure 1, uses both variables and outcomes. Could this approach introduce bias since the outcome is part of the imputation process? The authors should elaborate on this and justify their use of the GkNN method.

Regarding model explainability, SHAP should be elaborated upon in the methods section to explain why it was used and its advantages.

The format of Table 1 appears non-standardized. The authors are advised to carefully calculate the numbers and report them accurately. A standard “Table 1” format should be considered. For reference, see the example here:
https://cran.r-project.org/web/packages/table1/vignettes/table1-examples.html

In Table 2, the 95% confidence intervals are missing. Furthermore, the cut-off values for calculating sensitivity, etc., are absent.

Figure 3: As mentioned earlier regarding the use of SHAP, while variable importance is one of its features, it is not the only one. The ranking may provide certain insights into the model, but the authors are encouraged to explore other components of SHAP to help readers interpret the models and their decision-making processes. 

Decision letter
Manuscript ID OXFNSC-2024-003 entitled "Machine Learning-Based Prediction of One-Year Mortality in Ischemic Stroke Patients" which you submitted to the Oxford Open Neuroscience, has been reviewed. Thank you for your patience while we considered this manuscript. The comments of the reviewer(s) are included at the bottom of this letter.

Please note that this journal operates with transparent peer review. This means that if your submission is accepted for publication, the full peer review history of your article will publish online alongside your article. This includes reviewer comments, editor decision letters, and your author responses.

The reviewer(s) appreciated the merits of this manuscript, but also suggested some major revisions to your manuscript.  Therefore, I invite you to respond to the reviewer(s)' comments and revise your manuscript within the next 60 days from today date.

Authors’ response
Dear Editor,
We sincerely appreciate all the valuable suggestions and comments, which assisted us in improving the quality of our manuscript. Our response to the reviewers’ comments is described below in a point-to-point manner. Appropriated changes suggested by the reviewers have been introduced to the manuscript.
Reviewer: 1

Comment: Although the approach is novel, the results are mostly expected.
Answer: That is an insightful comment that precisely describes why AI enthusiasts recognize the immense potential of machine learning. Machine learning can achieve results comparable to traditional scientific methods but in a faster and significantly more cost-effective manner. This strengthens the argument that machine learning is invaluable in advancing medical sciences by leveraging the vast amounts of data already available in medical databases to generate meaningful evidence, which has the potential to change the way evidence is generated in the future.

Comment: The only exception is smoking which is a known stroke risk factor. The patients included is possibly a reason for the outcome. There are 80% male stroke patients which is different from the global data with fewer male cases than female cases. Qatar has a large population of migrant workers. Most of them are young male. This group of patients may lead to a younger age of stroke, usually with a better outcome. It is possible this group of patients has a higher rate of smoking.
Answer: Thank you for the comment. The 'smoking paradox' is indeed well-documented in stroke and other chronic disease research. We agree that various factors—such as data quality and population age—could contribute to this phenomenon and numerous other potential influences. In our analysis, we aimed to provide a contextual explanation centered around age differences, supported by benchmarks from previous research findings

Comment: Also there is a lack of discussion on thrombolysis and thrombectomy treatment on prognosis. These are important treatment for ischemic stroke. Even without a significance, there is a need to discuss the potential mechanisms.
Answer: Thank you for the comment. While we agree in principle, our strategy prioritized the most impactful variables to optimize model performance, avoiding an exhaustive discussion of all study variables, which would be impractical. Notably, thrombolysis and thrombectomy had less influence, and including every variable would risk diluting the paper's focus
Reviewer 2
Comment: In the Background section, the authors mentioned that scholars have developed and used models such as APACHE and SOFA to estimate the likelihood of stroke mortality. This statement requires adjustments, as neither score was specifically designed for stroke risk prediction.
Answer: Thanks for this observation. Corrected accordingly. (line 71)

Figure 1 requires further clarification. The links between several modules need to be clearly indicated and connected. For example, both training and test data are fed into the machine learning models; however, test data should be independently evaluated and therefore should be connected to model evaluation. Additionally, SHAP analysis is incorrectly linked to model comparison. SHAP is a post-hoc explanation tool that is separate from model comparison. The authors are encouraged to carefully examine the entire analytics pipeline to accurately reflect the work done.
Answer: Thank you for the constructive feedback. The figure is designed to provide a straightforward overview of the workflow from data acquisition to deployment, aiming to be accessible to non-experts by avoiding overly technical language. We split the data into training and test sets in developing the predictive model. The algorithms process these subsets sequentially—first by training on the training set, then by applying the learned parameters to the test set. The test set performance metrics (e.g., accuracy, precision, F-score) thus reflect the model's capability following training. Importantly, this process is embedded in the pipeline rather than involving two distinct and separately processed data sets.
We acknowledge that SHAP, as a post-hoc explanation tool, is independent of model performance metrics and is utilized here to explain variable contributions. In our workflow, after selecting the best-performing model, SHAP helped us interpret the importance of individual variables by ranking their impact on predictions. We hope this clarifies our stepwise approach and rectifies any potential confusion.

Comment: A major concern in the methods section is the lack of clarity regarding the timestamps on variables available for prediction. To predict one-year mortality post-stroke, what is the time point of prediction? What is the timeframe for variable collection, e.g., pre-stroke or post-stroke within a specified number of months? Without a clear definition, there is a risk that some patients might use data from the 11th month post-stroke to predict one-year mortality (i.e., at the 12th month), while others may have only one month of post-stroke data available. The study design should be standardized and elaborated upon.

Answer: Thank you for raising this valuable point. As explained in the ‘Outcome Variable’ subsection, our analysis focuses on mortalities within one year of admission, capturing 302 cases. The admission date serves as the reference point (Day 0) for ischemic stroke, as recorded in the registry. Patients undergo follow-up visits at 90 days, 6 months, and one-year post-discharge, with additional visits from the home care team as needed. This structure allows for a maximum interval of 6 months between data collection points, well within the one-year boundary. While we acknowledge the possibility of data gaps for some patients, we are fortunate to have robust data for the vast majority of ischemic stroke patients included in this study. An additional explanation is added to the “outcome variable” subsection.

Comment: Missing value imputation, as shown in Figure 1, uses both variables and outcomes. Could this approach introduce bias since the outcome is part of the imputation process?
Answer: Thank you for your comment. We did not impute the outcome variable; only input variables deemed valuable for the outcome, based on prior research, were imputed. While this approach is widely used, it does present challenges to the robustness of the prediction system, which we have addressed in detail in the limitations section.
Comment: The authors should elaborate on this and justify their use of the GkNN method.
Answer: The elaboration is done.

Comment: Regarding model explainability, SHAP should be elaborated upon in the methods section to explain why it was used and its advantages.
Answer: Addressed as suggested.

Comment: The format of Table 1 appears non-standardized. The authors are advised to carefully calculate the numbers and report them accurately. A standard “Table 1” format should be considered. For reference, see the example here:
https://cran.r-project.org/web/packages/table1/vignettes/table1-examples.html

Answer: Table 1 has been revised as suggested.

Comment: In Table 2, the 95% confidence intervals are missing. Furthermore, the cut-off values for calculating sensitivity, etc., are absent.
Answer: Thank you for this valuable comment. We strive to align the presentation of our results with established practices in the field, ensuring consistency with most prior work. Additionally, while we understand the importance of detail, including extensive information that may not significantly impact the interpretation of the numbers could risk overcrowding the table and reducing clarity for readers.

Comment: Figure 3: As mentioned earlier regarding the use of SHAP, while variable importance is one of its features, it is not the only one. The ranking may provide certain insights into the model, but the authors are encouraged to explore other components of SHAP to help readers interpret the models and their decision-making processes.

Answer: That is absolutely correct. However, additional features that SHAP offers, such as the SHAP violin plot, add limited value to our paper's objectives. The primary benefit researchers derive from SHAP is its ability to highlight feature importance, allowing for a more precise explanation of each variable's contribution to the prediction model. This aspect has been thoroughly addressed and discussed. While the violin plot provides insight into the directionality of relationships between input and output variables, we conveyed this information through a comprehensive explanation of these relationships in the discussion section. There, we included secondary analyses (e.g., correlation, association, t-test, p-value, etc.) to present the relationships in a way that is more accessible and relevant to our audience. In other words, our paper focuses less on the technical details and more on delivering valuable clinical insights.

Final decision letter
It is a pleasure to accept your revised manuscript entitled "Machine Learning-Based Prediction of One-Year Mortality in Ischemic Stroke Patients" in its current form for publication in the Oxford Open Neuroscience.  The comments of the reviewer(s) who reviewed your manuscript are included at the foot of this letter.

Please note that this journal operates with transparent peer review. This means that  the full peer review history of your article will publish online alongside your article. This includes reviewer comments, editor decision letters, and your author responses.

Next steps
You will receive an email from no-reply@scipris.com within roughly one week. This is your invitation to sign up for an account with SciPris, Oxford University Press’ author portal hosted by Aptara. You will need to create an account if you do not already hold one. Please register or log into your account and follow the online instructions which will guide you through signing your licence and paying the APC. The email and the portal have clearly signposted support options if you need any help during this process.

Please note that SciPris is a completely different system from ScholarOne, so your credentials to submit your manuscript here will not work there. Once you’ve created a SciPris account, you will be able to use it whenever you publish with Oxford Open Neuroscience or any OUP journal. Please note that OUP will only ever request payment for applicable fees be made via SciPris or to an OUP bank account. If you ever have concerns about the legitimacy of a request, please do not hesitate to contact a customer services agent via the SciPris portal or directly via oupsupport@scipris.com.

Thank you for your fine contribution.  On behalf of the Editors of the Oxford Open Neuroscience, we look forward to your continued contributions to the Journal.
